# Antibiotic susceptibility pattern of Portuguese environmental Legionella isolates

**DOI:** 10.3389/fcimb.2023.1141115

**Published:** 2023-04-21

**Authors:** Carolina Cruz, Lúcia Rodrigues, Filipa Fernandes, Ricardo Santos, Paulo Paixão, Maria Jesus Chasqueira

**Affiliations:** ^1^ NOVA Medical School, Faculdade de Ciências Médicas, Universidade NOVA de Lisboa, Lisboa, Portugal; ^2^ Comprehensive Health Research Center, NOVA Medical School, Faculdade de Ciências Médicas, Universidade NOVA de Lisboa, Lisboa, Portugal; ^3^ Laboratório de Análises de Água, Técnico Lisboa, Universidade de Lisboa, Lisboa, Portugal

**Keywords:** *Legionella*, broth microdilution, MIC, fluoroquinolones, macrolides, environmental, antibiotic susceptibility

## Abstract

**Introduction:**

Legionnaires’ Disease is a pneumonia caused by *Legionella* spp., currently treated empirically with fluoroquinolones and macrolides. In this study, we aim to describe the antibiotic susceptibility pattern of environmental *Legionella* recovered in the south of Portugal.

**Methods:**

Minimal inhibitory concentration (MIC) determination of 57 *Legionella* isolates (10 Lp sg 1, 32, Lp sg 2-14 15 L. spp) was achieved by broth microdilution, as described by EUCAST, for azithromycin, clarithromycin, ciprofloxacin, levofloxacin, and doxycycline.

**Results:**

Fluoroquinolones were the most active antibiotic, displaying the lowest MIC values in contrast to doxycycline which had the highest. MIC90 and epidemiological cut-off (ECOFF) values were, respectively, 0.5/1 mg/L for azithromycin, 0.125/0.25 mg/L for clarithromycin, 0.064/0.125 mg/L for ciprofloxacin, 0.125/0.125 mg/L for levofloxacin and 16/32 mg/L for doxycycline.

**Discussion:**

MIC distributions were higher than reported by EUCAST for all antibiotics. Interestingly, two phenotypically resistant isolates with high-level quinolone resistance were identified. This is the first time that MIC distributions, *lpeAB* and tet56 genes have been investigated in Portuguese environmental isolates of *Legionella*.

## 1 Introduction

Legionnaires’ Disease (LD) is a pneumonia, often severe, caused by waterborne pathogens of the *Legionella* genera. Despite more than 20 of the 65 known *Legionella* species being able to cause human infection (Chambers et al., 2021), *Legionella pneumophila* (*Lp*) alone is responsible for more than 90% of reported cases (European Centre for Disease Prevention and Control, 2022). Transmission to human hosts occurs primarily through aerosolized water particles, with only one description of person-to-person transmission of LD (Correia et al., 2016). *Legionella* is ubiquitous in water, colonizing natural and artificial environments, such as cooling systems, air conditioners and water supply networks.

The antibiotics used in LD treatment include fluoroquinolones, macrolides, and tetracyclines, given their high cellular penetration, azithromycin, and levofloxacin being considered the first line (Mandell et al., 2007; Woodhead et al., 2011). However, cases of unresolved LD or with slow treatment have already been described. Bruin et al., described one case of susceptibility loss to ciprofloxacin treatment, derived from a mutation in position 83 of the *gyrA* gene which encodes a subunit of the DNA gyrase (Bruin et al. ). Additionally, resistance by target mutation has been comproved *in vitro* for macrolides and rifampicin (Nielsen et al., 2000; Descours et al., 2017).

Recently, other resistance mechanisms were reported in this bacterium associated with the LpeAB operon, an analog of the AcrAB efflux pump of *Escherichia coli* (Massip et al., 2017). Mutations upstream of *lpeAB* gene, have been described as responsible for a decreased susceptibility to azithromycin and erythromycin in *Lp* Paris strain. This LpeAB efflux pump has been found in clinical and environmental isolates (Vandewalle-Capo et al., 2017; Nata and Løva, 2019; Cocuzza et al., 2021). Tet56, a tetracycline destructase of the TetX family has been identified in *L. longbeacheae*, *L. nautarum*, and *L. jordanis* isolates (Forsberg et al., 2015; Joseph et al., 2016). Although the presence of Tet56 may not be clinically impactful, as tetracyclines are regarded as an alternative in LD treatment, tigecycline, a synthetic tetracycline, has been successfully used in cases of LD, when treatment with first-line antibiotics has failed (Valve et al., 2010; Slawek et al., 2017).

Antibiotic pollution is a worrying problem. The presence of antibiotics in different aquatic environments, usually in low concentrations (Hughes et al., 2013; Danner et al., 2019; Rodriguez-Mozaz et al., 2020), is known to influence resistance mechanisms (Andersson and Hughes, 2014; Murray et al., 2018; Chow et al., 2021), promote virulence factors (Andersson and Hughes, 2014), and decrease antibiotic susceptibility (Sulyok et al., 2017). However, the full extent of the impact caused by chronic exposure remains unknown (Janecko et al., 2016). As the source of LD infection is from water and soils, special concern should be given to environmental *Legionella* populations.

The high efficacy of LD antibiotic treatment, over reliance on urinary antigen tests and reduction in culture, and the lack of consensus in antimicrobial susceptibility testing (AST) methodology, are contributing factors to the absence of standardization, and clinically defined breakpoint (Portal et al., 2021a). In this study, we aim to characterize Portuguese environmental *Legionella* susceptibility patterns, define ECOFF, and contribute more data on the subject.

## 2 Methods

### 2.1 Bacterial isolates

Isolates were collected (n = 57) from November 2021 to June 2022. They were obtained from water samples screened for *Legionella* presence in LAIST (*Laboratório de Análises de Água, Técnico Lisboa, Universidade de Lisboa*), in accordance with the standardized procedures described in the ISO 11731 (International Organization for Standardization, 2017). All isolates were obtained from aerosol producing equipment. Isolates were discriminated into three groups (*Lp* sg 1, *Lp* sg 2-14, *L*. spp) based on serological identification with latex agglutination test (OXOID, UK). Bacterial isolates were then preserved at -80°C in a thioglycolate medium until tested.

### 2.2 Control strains

In this study, the control strains *Lp* sg1 ATCC 33152 and Paris was used.

### 2.3 Antibiotic agents

Five antibiotics were selected to be studied: azithromycin (AZT); clarithromycin (CLA); ciprofloxacin (CIP); levofloxacin (LEV) and doxycycline (DOX) [Sigma Aldrich, USA]. Antibiotics were resuspended in the advised solvents, respectively: methanol, DMSO, HCl 1% solution, and water for the last two. The range of concentrations tested were respectively: 0.016 – 4 mg/L; 0.004 – 2 mg/L; 0.016 – 1 mg/L; 0.008 – 0.5 mg/L; and 0.5 – 128 mg/L.

### 2.4 Susceptibility tests

Tests were performed in accordance with European Committee on Antimicrobial Susceptibility Testing (EUCAST) and Clinical and Laboratory Standards Institute (CLSI) guidelines (Clinical and Laboratory Standards Institute, 2018; European Committee on Antimicrobial Susceptibility Testing, 2021). Briefly, bacterial suspensions of 0.5 McFarland were prepared using buffered yeast extract medium (BYE). Antibiotics were diluted in ultrapure water in ranges equal to the minimal inhibitory concentration (MIC) distribution of EUCAST (European Committee on Antimicrobial Susceptibility Testing, 2021). Forty microliters of each antibiotic dilution were added to the test wells of the microplate before adding the 160 µL of the bacterial suspension. For positive and negative controls bacterial suspension and BYE medium were used, respectively. All isolates were tested in duplicate.

Microplates were incubated at 37°C for 48 hours in a humid chamber. After incubation, the microplates were read manually according to by EUCAST and CLSI guidelines. Additionally, absorbances were read in a spectrophotometer at a wavelength of 600 nm and the inhibition rate was calculated using the formula:


$$IR=\;100-\left(\frac{Abs_{x}-Abs_{C-}}{Abs_{C+}-Abs_{C-}}*100\right)$$

Where Abs_X_ represents the absorbance of the sample; Abs_C-_ and Abs_C+_ the absorbance of the negative and positive control, respectively; IR the inhibition rate. To determine MIC a cut-off value of 90% inhibition was used.

### 2.5 Definition of the wild-type Portuguese ECOFF

ECOFFs were defined through the ECOFFinder program available at the EUCAST website, that follows the methodologies described by Turnidge et al. (Turnidge et al., 2006; European Committee on Antimicrobial Susceptibility Testing, 2023).

### 2.6 Molecular detection of resistance mechanisms

Molecular amplification of *lpeAB* and *tet56* genes was performed through conventional PCR to determine the presence of possible resistance mechanisms. Briefly, for the gene *lpeAB* the specific primers lpp2879_detec_F2 (5′-GTGATGATTGTCTTATTGG TGCGA-3′) and lpp2879_detec_R3 (5′-ATGGCGTTTAAGATGATGGT GATT-3′) were used (Vandewalle-Capo et al., 2017) and, for the gene *tet56* the primers Fw (5’- ATGTCTAAAAATATCAAAATTCTCGTC-3’) and Rv (5’- CTATGATGATTCATATTGAGGTAAGG-3’) (Forsberg et al., 2015). The presence of the *lpeAB* gene was investigated in all isolates tested for azithromycin. The presence of the *tet56* gene was investigated in *Lp* sg1 and *Lp* sg 2-14 isolates when MIC for doxycycline was raised to EUCAST tentative highest MICs, 2 mg/L and 32 mg/L respectively, and in all *L*. spp isolates.

### 2.7 Statistical analysis

To compare the two reading methods, the MIC distributions between serological groups from the present study to the EUCAST values and with the MICs values described in selected studies (Wilson et al., 2018; Assaidi et al., 2020; Cocuzza et al., 2021), non-parametric Mann-Whitney and Kruskal-Wallis test were performed using XLSTAT Software (Addinsoft, France).

## 3 Results

Thirty eight of the collected isolates were from water distribution systems of hospitals, hotels, and other public spaces, two isolates were from industrial cooling towers, one came from residual waters and, the remaining were of unknown origin. Water samples were collected from the southern region of Portugal, mainly from the Lisbon metropolitan area and Alentejo. In the studied population the three groups are represented: *Lp* sg 1 (n = 10), *Lp* sg 2-14 (n = 32), and *L.* spp (n = 15).

MIC distributions and ECOFFs are shown in **Table 1**. Overall, both quinolones achieved the lowest ECOFF values: 0.125 mg/L for both CIP and LEV; 0.25 mg/L for CLA; 1 mg/L for AZT; and 32 mg/L for DOX, which is the highest ECOFF.

**Table 1:** Cumulative percentages of MIC distribution of environmental *Legionella* isolates (n = 57) obtained by manual and automated reading (signalized with *).

	MIC distributions (mg/L)													
	0.008	0.016	0.032	0.064	0.125	0.25	0.5	1	2	4	8	16	32	64
AZT				4	28	68	93	98	100					
CLA	7	9	19	74	96	96	98							100
CIP			56	88	93	96							100	
LEV		23	70	89	96				98			100		
DOX								2	14	32	63	95	98	100
AZT*					21	33	86	96	100					
CLA*	2	12	19	44	70	89	96	98^+^						
CIP*		2	49	77	89	91	96						98^+^	
LEV*		19	63	75	91	96				98		100		
DOX*								2	7	16	30	72	95	100

+) For two isolates it was not possible to determine the MIC for the automated reading of CLA and CIP, respectively. T, Azithromycin; CLA, Clarithromycin; CIP, Ciprofloxacin; LEV, levofloxacin; DOX, Doxycycline.ECOFFs are represented in grey shading.

Comparison of MIC distributions between serological groups (**Table 2**) showed that *Lp* sg 1 had the highest MIC values. The ranges of the *Lp* sg 2-14 and *L.* spp groups were identical for LEV. For CIP the *Lp* sg 2-14 group achieved the lowest MIC range and for the other antibiotics, the *L.* spp group had the lowest range. Generally, the groups followed the above-described trend in MICs, except for CIP and CLA in the *Lp* sg 1 and *L.* spp groups, as in these, CLA presented a lower MIC 90 and range than CIP.

**Table 2:** MIC_50_, MIC_90_, and range values of five antibiotics for segregated serological groups of 57 environmental isolates obtained by manual and automated reading (within parenthesis).

	*Lp* sg 1 (n=8)*			*Lp* sg 2-14 (n=32)			*L*. spp (n=15)			Control Strains	
	MIC 50	MIC 90	Range	MIC 50	MIC 90	Range	MIC 50	MIC 90	Range	ATCC *Lp.* 33152	*Lp.* Paris
AZT	0.25 (0.5)	0.5 (1)	0.064 – 0.5 (0.064 - 1)	0.25 (0.5)	0.5 (0.5)	0.125 – 1 (0.064 - 2)	0.25 (0.5)	0.5 (0.5)	0.064 - 0.5 (0.064 - 1)	0.125 (0.125)	0.25 (0.125)
CLA	0.064 (0.064)	0.064 (0.125)	0.032 – 64 (0.032 – 0.125)	0.064 (0.125)	0.125 (0.25)	0.008 - 0.5 (0.016 - 1)	0.064 (0.064)	0.064 (0.5)	0.008 - 0.125 (0.008 – 0.5)	0.032 (0.032)	0.064 (0.064)
CIP	0.032 (0.064)	0.125 (0.125)	0.032 – 32 (0.032 – 0.125)	0.032 (0.032)	0.064 (0.064)	0.032-0.125 (0.032 – 0.125)	0.032 (0.064)	0.25 (0.5)	0.032 - 0.25 (0.016 – 0.5)	0.032 (0.064)	0.032 (0.064)
LEV	0.032 (0.032)	0.032 (0.064)	0.016 – 0.032 (0.032 – 0.064)	0.032 (0.032)	0.064 (0.125)	0.016-0.125 (0.016 – 0.125)	0.032 (0.032)	0.064 (0.25)	0.016 - 0.125 (0.016 – 0.25)	0.016 (0.032)	0.032 (0.125)
DOX	4 (16)	16 (32)	2 – 16 (8 - 32)	8 (16)	16 (32)	1 – 32 (1 - 64)	4 (8)	8 (32)	2 – 16 (2 - 64)	4 (2)	2 (16)

MIC values are presented in mg/L.

*This group excludes the two phenotypical resistant isolates.

Regarding MIC 50, values were similar in all groups for all antibiotics except for DOX in the *Lp* sg 2-14. Contrarily for MIC 90, values do not follow any trend in the three groups (**Table 2**).

A closer analysis of discriminatory MIC 50 and MIC 90 values demonstrated that the presence in two isolates showed a disequilibrium in these parameters. These discrepancies were caused by two phenotypically resistant strains (**Table 1**, **supplementary material S1**). Both isolates were collected from a hotel water system. The results of these two isolates have been retested in triplicate. These two isolates were also investigated for mutations in the QRDR of gyrA and no mutations were found in positions 83 and 87 (E. coli numbering, **Table S3** and **Figure S1**), suggesting a resistance mechanism different from one starting at GyrA position 83 (Almahmoud et al., 2009).

MIC distributions obtained through manual and absorbance readings were revealed to have statistically significant differences (p-value< 0.05). For all serological groups and antibiotics tested, the absorbance reading method produced higher MICs. Isolates *Lp* sg 1 and *Lp* sg 2-14 showed similar MIC in both methods, except for DOX and CLA. The MICs of *L.* spp were at least twice as high, except for AZT. DOX was the antibiotic that revealed the greatest discrepancy between the reading methods (**Table 3**).

**Table 3:** Ratios comparing automatic to the manual reading of MICs discriminated by serological group of isolates and antibiotic.

Groups	AZT	CLA	CIP	LEV	DOX
*Lp* sg 1 (n=10)	1,60	1,76	1,66	1,50	5,60
*Lp* sg 2-14 (n=32)	1,71	2,19	1,29	1,17	1,74
L spp (n=15)	1,67	2,49	2,27	2,26	3,20
All (n=57)	1,68	2,20	1,61	1,50	2,84

The results presented are the averages for each group. Statistical tests (Mann-Whitney and Kruskal-Wallis nonparametric test) were previously used to determine that the results obtained from manual reading and automated reading were statistically different. Statistical differences described elsewhere (**Table S4**).

**Figure 1:**
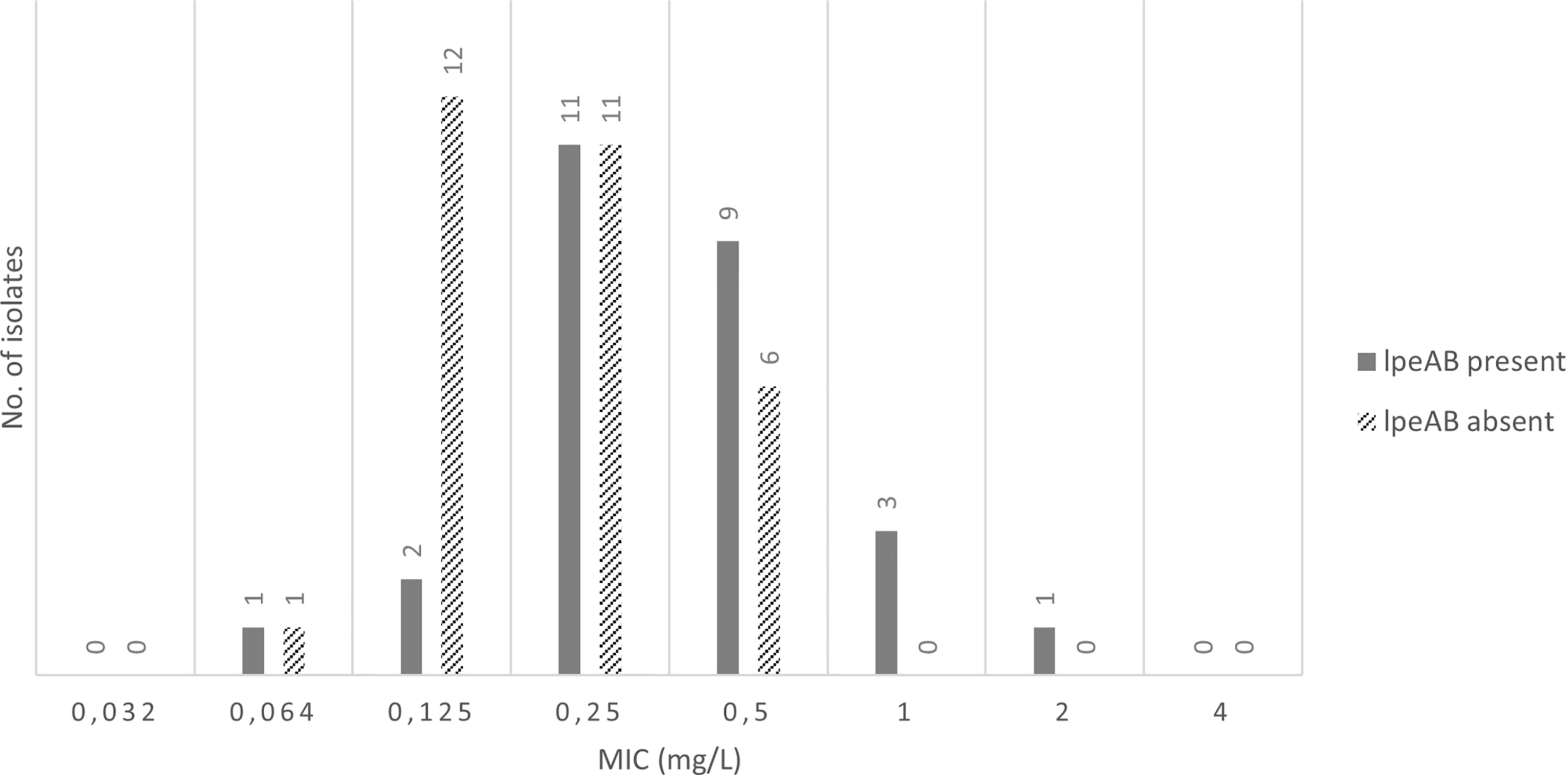
Presence of *IpeAB* gene and its distribution according to the AZT MIC of the 57 environmental isolates under study.

The evaluation results of the efflux pump gene presence showed that 67% (6/9) of the *Lp* sg 1 isolates and 60% (12/20) of the *Lp* sg 2-14, with MIC values, for AZT, greater than 0.125 mg/L, revealed the presence of the efflux pump gene, as well as 53% (8/15) of the studied *L.* spp. Overall, the *lpeAB* gene was present in 62% (18/29) of *Lp* sg 1 and *Lp* sg 2-14 isolates analyzed. The results showed that the number of isolates without the *lpeAB* gene was more prevalent for MICs equal to or less than 0.250 mg/L. Conversely, for higher MICs, isolates with this gene predominate (**Figure 1**, **supplementary material S2**).

Converse to the presence of the *lpeAB* gene, the *tet56* gene was found in only one *L.* spp isolate of those analysed (1/21) and identified by MALDI-ToF as *Legionella anisa*.

In summary, the achieved results did not show significant differences comparing the MIC distributions in the three groups, except the MIC distributions of CLA and DOX in *Lp* sg 2-14 and *L*. spp. MIC distributions obtained manually and automatedly were found to be significantly different (p-value< 0.05). By comparison, our MIC distributions is statistically different from EUCAST and selected studies, Cocuzza et al. (Cocuzza et al., 2021); Assaidi et al. (Assaidi et al., 2020); Wilson et al. (Wilson et al., 2018), except for AZT where similar distributions are found (**Table S4**).

## 4 Discussion

In 1999 LD was included for mandatory clinical and laboratory notification as part of the Portuguese infectious disease surveillance scheme. The disease is potentially life-threatening if not treated with the proper antibiotics.

Environmental monitoring of *Legionella*, in our country, became mandatory after the publication of Law n. 52/2018, of August 20 (Law n.o 52/2018, of Aug 20, 2023). The Law proposed periodic sampling of water from heat transfer equipment associated with heating, ventilation, air systems, air conditioning units, or air treatment, which produce water aerosols. However, in our study, most of the isolates were recovered from water distribution systems in hospitals and hotels. Growth inhibition, due to a high concentration of disinfectant or another factor, could be a plausible explanation for this lower number of isolates from heat transfer equipment (García et al., 2007; Sanli, 2019).

Regarding the serological distribution, the predominant group was *Lp* sg 2-14, which matches with previous studies (Xiong et al., 2016; Graells et al., 2018; Torre et al., 2018; Assaidi et al., 2020; Cocuzza et al., 2021; Zhan et al., 2022).

*Legionella* susceptibility analysis is important to predict the evolution of antibiotic resistance and assess its impact on the treatment of LD. This topic has been the subject of several studies, and the results obtained show there is a need for standardization of methodologies, and the establishment of guidelines for all groups of *Legionella* isolates to optimize the detection of resistances. In this study, susceptibility against five antibiotics was evaluated in 57 environmental isolates, and our results corroborate the trend in the effectiveness already reported, with floroquinolones being the most effective antibiotic and tetracyclines the least effective (Xiong et al., 2016; Vandewalle-Capo et al., 2017; Wilson et al., 2018; Assaidi et al., 2020; Cocuzza et al., 2021).

In contrast, MIC 50 and MIC 90 values are not concordant with the literature, being higher for quinolones and macrolides (Vandewalle-Capo et al., 2017; Wilson et al., 2018; Cocuzza et al., 2021). Nonetheless, Xiong et al. (2016) obtained similarly high values for DOX in the *Lp* sg 1 group, and Assaidi et al. (2020), reported elevated MIC 50 and MIC 90 for all antibiotics compared to current guidelines, except for AZT (Xiong et al., 2016; Assaidi et al., 2020). In comparison with EUCAST’s values, our MIC 50 and MIC 90 are bigger in *Lp* sg 1 and *Lp* sg 2-14 for AZT and CIP and in LEV and DOX for *Lp* sg 1, CLA for *Lp* sg 1, and LEV and DOX for *Lp* sg 2-14 agreed with the EUCAST guidelines (European Committee on Antimicrobial Susceptibility Testing, 2021).

EUCAST guidelines do not describe any MIC distributions for *L.* spp, although two other studies and these studies were in agreement with our results (Xiong et al., 2016; Assaidi et al., 2020).

The distribution of our MICs proved to be statistically different from those presented by EUCAST and in selected studies for *Lp* isolates (Wilson et al., 2018; Assaidi et al., 2020; Cocuzza et al., 2021; European Committee on Antimicrobial Susceptibility Testing, 2021). The comparison was performed using only studies that employed EUCAST methodologies, but the differences showed how important it is to carry out this type of investigation to provide additional data on *Lp* susceptibility so that an ECOFF can be defined. The global trend of increasing resistance levels implies a reduction of antibacterial treatment effectiveness that could have worrying consequences.

MIC values obtained through automated reading are higher than manual, making a defined cut-off difficult when comparing both methods. Operator variability and subjectiveness can contribute to a lack of standardization and as most results are read manually, so the comparison of MICs values can be controversial in studies.

To the best of our knowledge, this is the first time two phenotypically resistant isolates have been reported with such elevated MIC values (**Table S1**). These isolates possess an elevated risk, given their diminished susceptibilities to several of the first line antibiotics. While the LD mortality rate typically does not surpass 10% (European Centre for Disease Prevention and Control, 2022), cases have achieved rates of nearly 30%, in hospital-related LD cases (Beauté, 2017). The effect on increased morbidity caused by strains with this high MIC is unknown. Curiously, presence of the efflux pump gene, *lpeAB* was found in both isolates as expected given their MIC values for AZT.

Presence of *lpeAB*, in our isolates, was high. Previous studies reported a 50% prevalence (Portal et al., 2021b), but no studies have reported the *lpeAB* gene outside of *L. pneumophila*. This is the first time resistance has been reported in non pneumophila *Legionella*. Given the similarities between the MIC distributions for *L*. spp, with and without *lpeAB*, it is difficult to determine a clear cut-off value to distinguish the wild-type and *lpAB* positive isolates. This is mainly due to the small population analyzed.

Overall, these results do not diverge from EUCAST. Apart from a single *Lp* sg 2-14 all remaining isolates from this group and *Lp* sg 1 did not change the EUCAST distribution for LpeAB-positive isolates. Previous studies have also reported the presence of the efflux pump gene in isolates whose MIC values for AZT are equal to or exceed 0.125 mg/L (Vandewalle-Capo et al., 2017; Nata and Løva, 2019; Portal et al., 2021b). However, the presence of *lpeAB-negative* isolates with elevated MIC values as high as 0.5 mg/L was also observed in this study, and in previous works (Cocuzza et al., 2021; Yang et al., 2022). In this situation defining an ECOFF is a complex question given that the purpose is to discriminate between wild-type isolates and isolates containing acquired resistance mechanisms, and the efflux pump LpeAB has been described as an acquired resistance mechanism (Turnidge et al., 2006). Therefore, additional studies are required to provide robust ECOFF values.

The *tet56* gene was detected in one isolate with a MIC of 2 mg/L. This gene codes for a tetracycline destructase, and was first described in *L. longbeacheae* (Forsberg et al., 2015).

## 5 Conclusion

This study reports the susceptibility patterns of environmental Portuguese isolates, whose MIC distributions surpass EUCAST reference values. We also describe two phenotypically resistant isolates with high-level floroquinolone resistance. It would be interesting to pursue further genotypical characterization of these isolates, in particular the *parC*, *gyrB, parE, rrl, rplD* and *rplV* genes.

The study highlights the presence of the efflux pump gene *lpeAB* in Portuguese isolates, and the presence of the *tet56* gene in a single *L. anisa* isolate which, to the best of our knowledge, is the first reported instance regarding presence of tetracycline destructase in this *Legionella* species.

The current epidemiologic trends of growing incidence of LD cases in several countries, allied with the global antibiotic resistance accentuates the task of establishing robust ECOFF values, clinical breakpoints, and a standardized methodology. The present research points to the current need to increase knowledge about environmental populations of *Legionella* to predict the potential emergence of antibiotic resistance in clinical isolates.

## Data availability statement

The original contributions presented in this study are included in the article/**Supplementary Material**. Further inquiries can be directed to the corresponding author.

## Author contributions

CC carried out the experiments, analyzed the data, and wrote the original draft manuscript. LR made data interpretations and revised and edited the manuscript. FF isolated *Legionella* and made data interpretations. RS collected the environmental samples and revised the manuscript. PP verified the data and revised the manuscript. MC designed the study, verified the data, and revised and edited the manuscript. All authors contributed to the article and approved the submitted version.
