# Corrigendum: Antibiotic susceptibility pattern of Portuguese environmental *Legionella* isolates

**DOI:** 10.3389/fcimb.2023.1272773

**Published:** 2023-09-20

**Authors:** Carolina Cruz, Lúcia Rodrigues, Filipa Fernandes, Ricardo Santos, Paulo Paixão, Maria Jesus Chasqueira

**Affiliations:** ^1^ NOVA Medical School, Faculdade de Ciências Médicas, Universidade NOVA de Lisboa, Lisboa, Portugal; ^2^ Comprehensive Health Research Center, NOVA Medical School, Faculdade de Ciências Médicas, Universidade NOVA de Lisboa, Lisboa, Portugal; ^3^ Laboratório de Análises de Água, Técnico Lisboa, Universidade de Lisboa, Lisboa, Portugal

**Keywords:** *Legionella*, broth microdilution, MIC, fluoroquinolones, macrolides, environmental, antibiotic susceptibility

## Error in Figure/Table

In the published article, there was an error in **Table 1** as published. Five lines are missing from the table. The corrected **Table 1** and its caption Cumulative percentages of MIC distribution of environmental Legionella isolates (n = 57) obtained by manual and automated reading (signalized with *) appear below.

**Table 1:** Cumulative percentages of MIC distribution of environmental *Legionella* isolates (n = 57) obtained by manual and automated reading (signalized with *).

	MIC distributions (mg/L)													
	0.008	0.016	0.032	0.064	0.125	0.25	0.5	1	2	4	8	16	32	64
AZT				4	28	68	93	98	100					
CLA	7	9	19	74	96	96	98							100
CIP			56	88	93	96							100	
LEV		23	70	89	96				98			100		
DOX								2	14	32	63	95	98	100
AZT*					21	33	86	96	100					
CLA*	2	12	19	44	70	89	96	98^+^						
CIP*		2	49	77	89	91	96						98^+^	
LEV*		19	63	75	91	96				98		100		
DOX*								2	7	16	30	72	95	100

+) For two isolates it was not possible to determine the MIC for the automated reading of CLA and CIP, respectively. T, Azithromycin; CLA, Clarithromycin; CIP, Ciprofloxacin; LEV, levofloxacin; DOX, Doxycycline.

ECOFFs are represented in grey shading.

The authors apologize for this error and state that this does not change the scientific conclusions of the article in any way. The original article has been updated.

