# Supplementary material for: Antibiotic susceptibility pattern of Portuguese environmental Legionella isolates
Source: Front Cell Infect Microbiol. 2023 Apr 21;13:1141115. doi: 10.3389/fcimb.2023.1141115 (PMC10160366; doi:10.3389/fcimb.2023.1141115)
Supplement: Supplementary file 1 [file DataSheet_1.docx]

Supplementary Material

Antibiotic susceptibility pattern of Portuguese environmental Legionella isolates.

**Carolina Cruz^1^, Lúcia Rodrigues^1^, Filipa Fernandes^2^, Ricardo Santos^2^, Paulo Paixão^1^, Maria Jesus Chasqueira^1^***

**^1^CHRC, NOVA Medical School, Faculdade de Ciências Médicas, Universidade NOVA de Lisboa, Lisboa, Portugal**

**^2^Laboratory of Analysis, Instituto Superior Técnico, Universidade de Lisboa, Lisboa, Portugal**

*** Correspondence:**

**Maria Jesus Chasqueira**

**NOVA Medical School**

**Campo Mártires da Pátria, 130**

**1169-056 Lisboa**

[**mjchasqueira@nms.unl.pt**](mailto:mjchasqueira@nms.unl.pt)

# Supplementary Data

Table S1. MIC values for all antibiotics, both reading methods and presence of *lpeAB* and *tet56* genes. Results presented in mg/L. MIC values are the average of duplicates for all isolates except IST2 & IST24, those are the the average of triplicates.

| Isolates | Sg. | Manual method | | | | | Automated method | | | | | Gene presence | |
| --- | --- | --- | --- | --- | --- | --- | --- | --- | --- | --- | --- | --- | --- |
|  |  | AZT | CLA | CIP | LEV | DOX | AZT | CLA | CIP | LEV | DOX | *lpeAB* | *tet56* |
| IST2 | 1 | 1 | 0,032 | 32 | 16 | 4 | 1 | 0,125 | IND | 16 | 32 | Yes | No |
| IST4 | 1 | 0,25 | 0,064 | 0,125 | 0,032 | 16 | 0,25 | 0,064 | 0,064 | 0,032 | 16 | No | No |
| IST24 | 1 | 2 | 64 | 32 | 2 | 64 | 2 | IND | 32 | 4 | 64 | Yes | No |
| IST25 | 1 | 0,5 | 0,032 | 0,064 | 0,032 | 4 | 1 | 0,064 | 0,032 | 0,064 | 32 | Yes | No |
| IST26 | 1 | 0,25 | 0,064 | 0,032 | 0,032 | 16 | 0,5 | 0,125 | 0,125 | 0,064 | 16 | Yes | No |
| IST30 | 1 | 0,064 | 0,064 | 0,032 | 0,016 | 16 | 0,064 | 0,032 | 0,032 | 0,032 | 16 | No | No |
| IST34 | 1 | 0,25 | 0,064 | 0,032 | 0,032 | 2 | 0,5 | 0,064 | 0,064 | 0,032 | 8 | Yes | No |
| IST35 | 1 | 0,25 | 0,064 | 0,032 | 0,032 | 2 | 0,5 | 0,064 | 0,064 | 0,032 | 16 | No | No |
| IST36 | 1 | 0,25 | 0,064 | 0,032 | 0,032 | 2 | 0,5 | 0,064 | 0,064 | 0,032 | 16 | No | No |
| IST39 | 1 | 0,5 | 0,064 | 0,032 | 0,032 | 2 | 1 | 0,125 | 0,064 | 0,064 | 32 | Yes | No |
| IST1 | 2-14 | 0,5 | 0,125 | 0,032 | 0,032 | 8 | 0,5 | 0,125 | 0,032 | 0,064 | 8 | Yes | NT |
| IST3 | 2-14 | 0,5 | 0,125 | 0,032 | 0,032 | 8 | 0,5 | 0,125 | 0,032 | 0,032 | 8 | No | NT |
| IST5 | 2-14 | 0,5 | 0,064 | 0,032 | 0,064 | 16 | 0,5 | 0,25 | 0,064 | 0,125 | 16 | No | NT |
| IST6 | 2-14 | 0,125 | 0,064 | 0,064 | 0,016 | 16 | 0,125 | 0,064 | 0,064 | 0,016 | 32 | No | NT |
| IST7 | 2-14 | 0,125 | 0,125 | 0,064 | 0,016 | 16 | 0,125 | 0,125 | 0,064 | 0,032 | 32 | No | NT |
| IST8 | 2-14 | 0,25 | 0,064 | 0,032 | 0,016 | 8 | 0,25 | 0,125 | 0,032 | 0,016 | 32 | Yes | NT |
| IST9 | 2-14 | 0,25 | 0,125 | 0,064 | 0,032 | 16 | 0,25 | 0,25 | 0,032 | 0,032 | 16 | No | NT |
| IST12 | 2-14 | 0,25 | 0,125 | 0,064 | 0,016 | 16 | 0,5 | 0,25 | 0,032 | 0,016 | 16 | Yes | NT |
| IST13 | 2-14 | 1 | 0,125 | 0,064 | 0,064 | 16 | 1 | 0,125 | 0,032 | 0,032 | 4 | Yes | NT |
| IST14 | 2-14 | 0,125 | 0,125 | 0,032 | 0,064 | 1 | 0,5 | 1 | 0,032 | 0,125 | 1 | No | NT |
| IST15 | 2-14 | 1 | 0,125 | 0,064 | 0,016 | 16 | 1 | 0,064 | 0,032 | 0,032 | 8 | Yes | NT |
| IST16 | 2-14 | 0,5 | 0,125 | 0,032 | 0,016 | 4 | 0,5 | 0,25 | 0,032 | 0,016 | 4 | Yes | NT |
| IST17 | 2-14 | 0,25 | 0,064 | 0,032 | 0,016 | 8 | 0,5 | 0,25 | 0,032 | 0,016 | 32 | No | NT |
| IST18 | 2-14 | 0,25 | 0,064 | 0,032 | 0,016 | 8 | 0,5 | 0,25 | 0,032 | 0,016 | 16 | No | NT |
| IST19 | 2-14 | 0,25 | 0,5 | 0,032 | 0,064 | 16 | 0,25 | 0,5 | 0,032 | 0,125 | 16 | No | NT |
| IST28 | 2-14 | 0,5 | 0,125 | 0,064 | 0,125 | 16 | 0,5 | 0,125 | 0,064 | 0,125 | 16 | No | No |
| IST29 | 2-14 | 0,125 | 0,064 | 0,032 | 0,032 | 8 | 0,125 | 0,25 | 0,064 | 0,032 | 16 | No | NT |
| IST31 | 2-14 | 0,125 | 0,064 | 0,032 | 0,032 | 16 | 0,5 | 0,25 | 0,064 | 0,064 | 32 | No | NT |
| IST32 | 2-14 | 0,125 | 0,064 | 0,032 | 0,032 | 8 | 0,5 | 0,25 | 0,125 | 0,064 | 32 | No | NT |
| IST33 | 2-14 | 0,25 | 0,064 | 0,032 | 0,064 | 16 | 0,25 | 0,064 | 0,032 | 0,032 | 8 | Yes | NT |
| IST37 | 2-14 | 0,25 | 0,125 | 0,032 | 0,125 | 16 | 0,5 | 0,125 | 0,064 | 0,125 | 16 | Yes | NT |
| IST38 | 2-14 | 0,25 | 0,125 | 0,064 | 0,125 | 16 | 0,5 | 0,125 | 0,032 | 0,064 | 16 | Yes | NT |
| IST40 | 2-14 | 0,125 | 0,008 | 0,064 | 0,064 | 32 | 0,5 | 0,016 | 0,064 | 0,032 | 64 | Yes | No |
| IST41 | 2-14 | 0,5 | 0,008 | 0,064 | 0,032 | 32 | 0,5 | 0,016 | 0,064 | 0,032 | 16 | Yes | NT |
| IST42 | 2-14 | 0,25 | 0,008 | 0,064 | 0,032 | 16 | 0,25 | 0,016 | 0,032 | 0,032 | 16 | Yes | NT |
| IST43 | 2-14 | 0,125 | 0,064 | 0,125 | 0,064 | 8 | 0,064 | 0,125 | 0,032 | 0,032 | 16 | No | NT |
| IST44 | 2-14 | 0,25 | 0,064 | 0,032 | 0,032 | 8 | 0,064 | 0,064 | 0,032 | 0,032 | 16 | Yes | NT |
| IST53 | 2-14 | 0,125 | 0,064 | 0,032 | 0,064 | 8 | 0,125 | 0,064 | 0,125 | 0,032 | 16 | No | NT |
| IST55 | 2-14 | 0,125 | 0,064 | 0,032 | 0,032 | 8 | 0,125 | 0,064 | 0,125 | 0,032 | 16 | No | NT |
| IST56 | 2-14 | 0,25 | 0,064 | 0,032 | 0,032 | 8 | 2 | 0,5 | 0,032 | 0,032 | 32 | No | NT |
| IST57 | 2-14 | 0,125 | 0,064 | 0,064 | 0,016 | 8 | 0,125 | 0,064 | 0,032 | 0,016 | 32 | No | NT |
| IST59 | 2-14 | 0,125 | 0,064 | 0,064 | 0,016 | 8 | 0,125 | 0,125 | 0,064 | 0,016 | 32 | No | No |
| IST20 | spp. | 0,5 | 0,064 | 0,064 | 0,064 | 8 | 0,5 | 0,064 | 0,5 | 0,125 | 16 | No | No |
| IST21 | spp. | 0,25 | 0,032 | 0,032 | 0,016 | 2 | 0,25 | 0,032 | 0,032 | 0,016 | 2 | Yes | Yes |
| IST22 | spp. | 0,5 | 0,064 | 0,064 | 0,032 | 2 | 0,5 | 0,125 | 0,125 | 0,25 | 16 | No | No |
| IST23 | spp. | 0,25 | 0,032 | 0,032 | 0,016 | 4 | 0,5 | 0,008 | 0,032 | 0,016 | 4 | No | No |
| IST27 | spp. | 0,5 | 0,064 | 0,064 | 0,032 | 4 | 0,5 | 0,125 | 0,125 | 0,125 | 64 | Yes | No |
| IST45 | spp. | 0,125 | 0,064 | 0,125 | 0,064 | 8 | 0,125 | 0,5 | 0,5 | 0,25 | 16 | No | No |
| IST46 | spp. | 0,25 | 0,064 | 0,032 | 0,032 | 8 | 0,5 | 0,064 | 0,064 | 0,032 | 8 | Yes | No |
| IST47 | spp. | 0,5 | 0,064 | 0,25 | 0,125 | 16 | 0,5 | 0,5 | 0,125 | 0,125 | 8 | Yes | No |
| IST48 | spp. | 0,25 | 0,064 | 0,032 | 0,032 | 4 | 1 | 0,016 | 0,25 | 0,125 | 16 | Yes | No |
| IST49 | spp. | 0,5 | 0,125 | 0,25 | 0,064 | 4 | 0,5 | 0,25 | 0,5 | 0,25 | 4 | Yes | No |
| IST50 | spp. | 0,125 | 0,032 | 0,032 | 0,032 | 4 | 0,5 | 0,25 | 0,032 | 0,032 | 32 | Yes | No |
| IST51 | spp. | 0,25 | 0,064 | 0,032 | 0,032 | 4 | 0,5 | 0,032 | 0,032 | 0,032 | 2 | No | No |
| IST52 | spp. | 0,25 | 0,016 | 0,064 | 0,032 | 4 | 0,5 | 0,016 | 0,032 | 0,032 | 4 | No | No |
| IST54 | spp. | 0,5 | 0,032 | 0,032 | 0,032 | 2 | 0,5 | 0,032 | 0,016 | 0,032 | 2 | No | No |
| IST58 | spp. | 0,064 | 0,008 | 0,032 | 0,032 | 8 | 0,064 | 0,016 | 0,032 | 0,016 | 8 | Yes | No |

IND – indeterminate result, as growth was visible in all concentrations tested.

NT – not tested, gene was not researched in this isolate.

Table S2. Presence of *lpeAB* and *tet56* genes in isolates with MIC values above EUCAST tentative highest MIC values, for *Lp* sg 1 and sg 2-14, and all *L.* spp isolates.

|  | *lpeAB* | | *tet56* | |
| --- | --- | --- | --- | --- |
|  | Positive | Negative | Positive | Negative |
| *Lp* sg 1 | 6 (67%) | 3 (33%) | 0 (0%) | 6 (100%) |
| *Lp* sg 2-14 | 12 (60%) | 8 (40%) | 0 (0%) | 0 (0%) |
| *L.* spp | 8 (53%) | 7 (47%) | 1 (7%) | 14 (93%) |


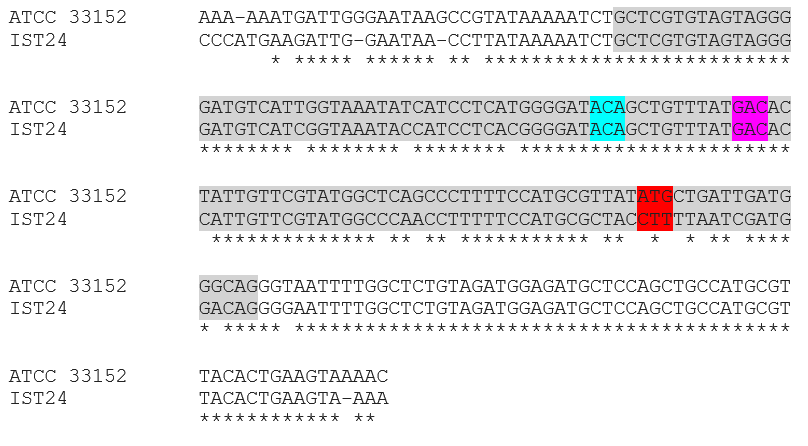

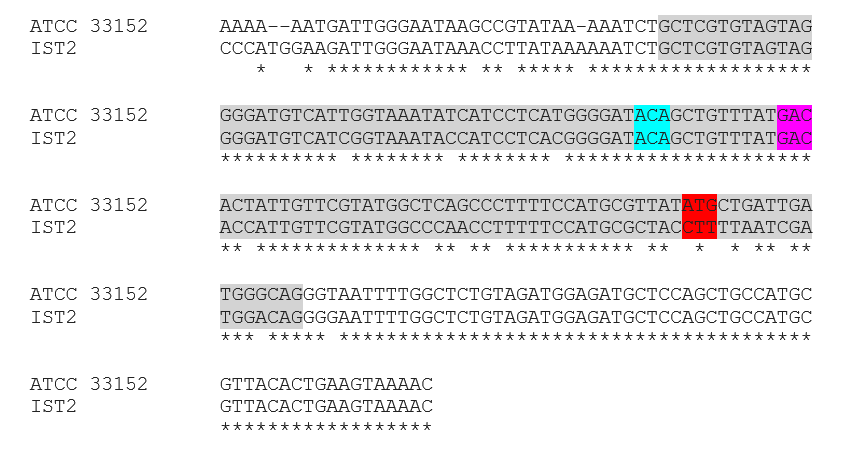

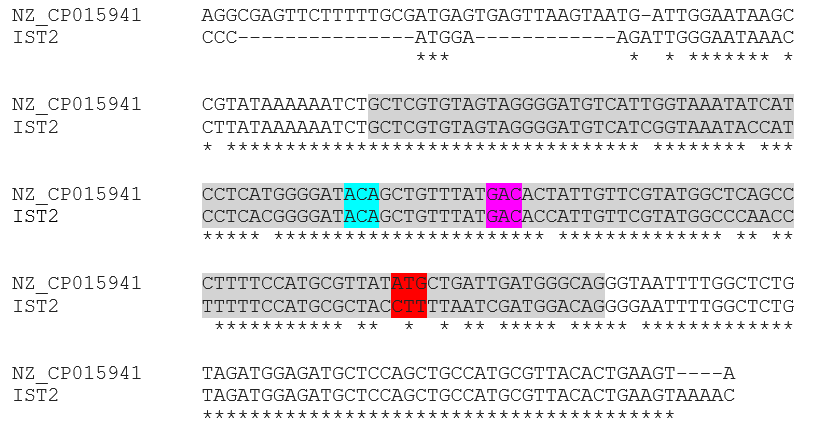


**D**

**C**

**A**

**B**


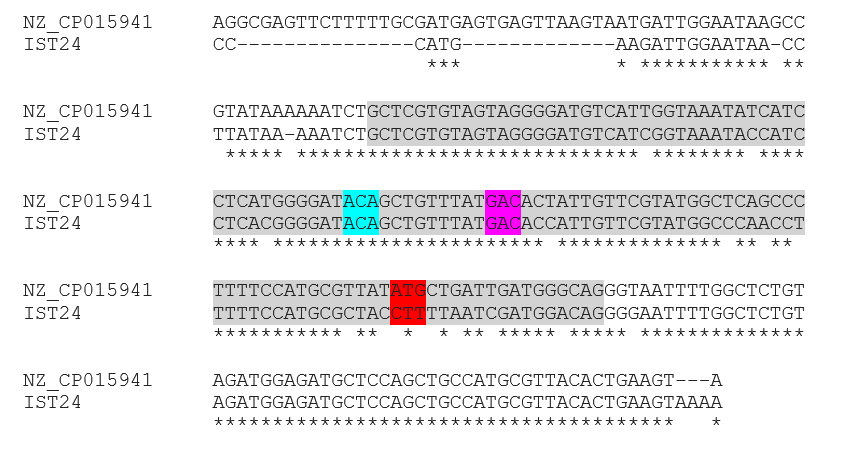


Figure S1. Alignment of *gyrA* QRDR sequences of the phenotypically resistant isolates (IST2 and IST24) identified in this study, using a multiple alignment sequence tool (T-Coffee). A and C are comparisons with GenBank sequence access number shown, B and D are comparisons with the control strain shown. GyrA codons at positions 83, 87 & 101 (E. coli numbering) are highlighted in the colors blue, pink and red, respectively.

| Position | Gene | | Protein | | Position | Gene | | Protein | |
| --- | --- | --- | --- | --- | --- | --- | --- | --- | --- |
|  | Control | IST2/IST24 | Control | IST2/IST24 |  | Control | IST2/IST24 | Control | IST27IST24 |
| 74 | ATT | ATC | Ile (I) | Ile (I) | 77 | TAT | TAC | Tyr (Y) | Tyr (Y) |
| 80 | CAT | CAC | His (H) | His (H) | 88 | ACT | ACC | Thr (T) | Thr (T) |
| 93 | GCT | GCC | Ala (A) | Ala (A) | 94 | CAG | CAA | Gln (Q) | Gln (Q) |
| 95 | CCC | CCT | Pro (P) | Pro (P) | 99 | CGT | CGC | Arg (R) | Arg (R) |
| 100 | TAT | TAC | Tyr (Y) | Tyr (Y) | **101** | **ATG** | **CTT** | **Met (M)** | **Leu (L)** |
| 102 | CTG | TTA | Leu (L) | Leu (L) | 103 | ATT | ATC | Ile (I) | Ile (I) |
| 105 | GGG | GGA | Gly (G) | Gly (G) |  |  |  |  |  |

Table S3. Mutations (in *E. coli* numbering) identified in the *gyrA* QRDR of the phenotypically resistant isolates (IST2 and IST24). Expressing mutations are in bold.

S4. P-values obtained from the comparison of our results to selected studies with Mann-Whitney Test. Comparisons with p-value > 0.05 were considered statistically similar.

|  | Cocuzza et al | Assaidi et al | Wilson et al | EUCAST |
| --- | --- | --- | --- | --- |
| AZT | <0,0001 | 0.355 | NC | <0,0001 |
| CLA | NC | <0,0001 | NC | <0,0001 |
| CIP | <0,0001 | <0,0001 | <0,0001 | <0,0001 |
| LEV | <0,0001 | <0,0001 | <0,0001 | <0,0001 |
| DOX | NC | <0,0001 | NC | <0,0001 |

NC – Not comparable. The selected study did not aboard this antibiotic.
